# "The Hospital" Nursing Mirror

**Published:** 1897-01-23

**Authors:** 


### "The Hospital" Nursing Mirror.

Being the Nursing Section of "The Hospital."

[Contributions for this Section of "The Hospital" should be addressed to the Editor, The Hospital, 28 & 29, Southampton Street, Strand, London, W.C., and should have the word "Nursing" plainly written in left-hand top corner of the envelope.]

### News from the Nursing World.

### VICTORIA HOSPITAL FOR CHILDREN.

H.R.H. Princess Louise (Marchioness of Lorne) was present at the entertainment given the other day to the little patients in the Victoria Hospital for Children, an institution in which the Princess takes much interest. Her Royal Highness was received by Miss Hamilton, the matron, and by the chairman, Mr. Martin R. Smith, and stayed for some time, going round the wards and talking to and hearing about the children in the cots. There were Punch and Judy shows, and a number of nice presents to be distributed amongst the children, who spent a very happy afternoon.

### SEASIDE CONVALESCENT HOME FOR MOTHERS.

In 1890 a Mothers' Convalescent Home was started at Herne Bay, and since then more than fifteen hundred poor women and their babies have derived good from the change, fresh air, and kindly nursing thus brought within their reach. They come from all parts of England, many being sent through the London hospitals and the Sunday Fund. Funds are wanted to add to the nursing accommodation, and to build a large airy room in the grounds for the children, and for this the treasurer, Sir Rawson W. Rawson, is especially appealing, the home being supported solely by its subscriptions, which hardly suffice to keep it in full work and free from debt. The Secretary at the Home, 15, St. George's Terrace, Herne Bay, will gladly answer any inquiries.

### DARWEN NURSING ASSOCIATION.

The Darwen District Nursing Association reports a very satisfactory year's work. The work of the nurses is much appreciated in the place, and the association has been so well supported that the accounts at the end of 1896 showed a balance in hand of £146 14s. 5d., as against £60 2s. 8d. at the beginning of the year. The income for the year amounted to £372 3s. 5d., of which no less than £166 4s. came from collections in the mills and factories. The committee have decided to engage one more maternity nurse.

### AT THE ALEXANDRA HOSPITAL.

A good many visitors were present at the Alexandra Hospital for Children with Hip Disease in Queen Square on January 14th, when an entertainment was got up for the pleasure of the little patients. The wards were made bright with flowers for the occasion, and the children's enjoyment of the toys and pretty things distributed amongst them was pleasant to see. There were also marionettes and exhibitions of conjuring and ventriloquising. This little hospital, the only one in London where children suffering from this terrible malady can be kept for the length of time sometimes necessary for its proper treatment, just now very urgently needs help. The old Bloomsbury mansion in Queen Square is quite unfit for the purposes of a hospital, and this thirtieth year of its existence, it is hoped, will see the commencement of a new building for which a sum of £8,000 is still required. To help in raising the money the Duke of Fife is to preside at the first festival dinner to be held on April 8th.

### "A GOOD IDEA."

In the Mirror of the 19th ultimo we called attention to an excellent way to celebrate Christmas time at St. Mary's Hospital, Paddington, by hiring a piano for a week for each ward, so that a series of pleasant concerts might delight the patients throughout the week at the most convenient hour of each day. We are glad to hear on authority that the idea emanated from the secretary, Mr. Thomas Ryan, who defrayed the whole of the cost out of the money placed at his disposal for the Christmas Entertainment Fund. Mr. Ryan's idea is an excellent one; it has been carried out free of all cost to the nurses. We congratulate him upon his energy and success, and hope that the example thus set may be followed in future years by secretaries of hospitals throughout the country.

### THE EIGHT HOURS DAY.

"A Few Objections to the Eight-Hour System" are well set forth by Mabel Morrison in a recent number of the *Trained Nurse.* The writer, besides pointing out the evil effect upon patients of "an infinite variety of questionable care," caused by the constant change of hand during the twenty-four hours, dwells upon another point overlooked by those who advocate the establishment of an eight-hours day for nurses. It is not to be expected that private patients will be prepared to follow this system in cases of illness in their own homes, and the nurse who takes up private work after leaving hospital would be less fitted than she is at present to cope with the difficulties and "hardships" of this branch of nursing. It is the present regrettable demand for a quite unnecessary amount of theoretical study which alone will be responsible for the future introduction of this system into general use, and at present, fortunately, the weight of competent opinion in England goes against it, while English nurses who have had practical acquaintance with the working of the eight-hours day in foreign hospitals hold strongly the same view.

### DARLINGTON NURSING ASSOCIATION.

The people of Darlington seem making up their minds to commemorate the present year by raising a fund in aid of the local Queen's Nursing Association. A satisfactory meeting of friends of this institution was held the other day by invitation of Mr. and Mrs. Pease, presided over by Sir David Dale, Bart., at which the Mayor was present, and the claims of the district nurses were eloquently put forward by Canon Body. The purchase of a new home for the nurses has lately been found necessary, and to clear off a debt incurred on this account money is specially needed at the present moment. The Darlington Association affiliated with the Queen's Jubilee Institute in 1892, the number of nurses having increased since then from one to four.

### BEATTY v. CULLINGWORTH.

At the Court of Appeal, on January 13th, before the Master of the Rolls and Lords Justices Lopes and Chitty, an application for a new trial was brought by the plaintiff in this case, against whom judgment was given by Mr. Justice Hawkins. The appeal was dismissed.

### DISTRICT NURSING IN CORNWALL.

The St. Agnes District Nursing Association is lamenting the resignation of Nurse Salt, who, during her connection with it, has made herself many friends among the poor and sick in the district. Good wishes and gratitude will go with her to a new sphere of work. The association is well remembered by St. Agnes settlers in South Africa, who have recently, for the second time, sent home from Krugersdorp a welcome contribution to its funds through Mrs. Johnson.

### WORKHOUSE NURSING AT TIVERTON.

The trained nurse is apparently little known to the Guardians of Tiverton, who seem to have a holy horror of introducing such an alarming person into their infirmary. At a recent meeting of the Board, Mrs. Wyndham brought forward a proposal for the appointment of "an experienced nurse and assistant nurse for Tiverton Workhouse Infirmary," stating that she did not want "a woman to be engaged who had to gain her experience on their poor subjects, nor was she in favour of a hospital nurse or one of those ladies they saw flaunting their fine strings, but what she wanted was a practical woman, holding, if possible, a certificate of midwifery." One Guardian agreed to the appointment of an "experienced" nurse, but objected to a "trained hospital nurse," as trained nurses "came into collision with matrons, and wanted a good deal of waiting on"; while another "thought an intelligent woman would in a short time get such experience in the workhouse infirmary wards as to gain a certificate of her own"—whatever that may mean. Ultimately it was decided to advertise for "an experienced nurse," at £25 a year, and an assistant nurse, at £20 a year.

### A WOMEN'S HOSPITAL AND ITS FOUNDERS.

There is a very interesting article on "The New England Hospital for Women and Children and its Incorporators" in the January number of *The Trained Nurse*, illustrated with portraits of the three founders —Miss Lucy Goddard, Mrs. Ednah Dow Cheney, and Dr. Marie Zakrzewska. Miss Goddard, its first president, is now over ninety years of age. All three are honoured names in America, where medical women owe much to the untiring labours of these pioneers.

### NURSES IN SHANGHAI.

The three English nurses at Shanghai are finding their services in very great request, and have met with much kindness from the English residents. They are established in very comfortable quarters, from which, however, they expect to move as soon as a house can be found suitable for taking in private patients. The want of a few beds for such private cases has been much felt. Miss Campbell writes that the appearance of nurses in uniform has created quite a sensation in the streets, no such garb having hitherto been seen in Shanghai.

### DISTRICT NURSING AT SUDBURY.

The Sudbury and Ballingdon District Nursing Association became affiliated with the Queen's Jubilee Institute about a year ago, and a great deal of interest has been taken in the whole scheme by the people of the neighbourhood. At the r ecent annual meeting, presided over by Lady Florence Barnadiston, much was said of the value of the nurse's work, the careful attention given to the sick poor, and the educational influence of the nurse's visits in their homes. During the past year sixty-eight patients were nursed, and 3,273 visits paid to them. The expenses amounted to £71 16s., and the receipts to £93 18s. 8d., leaving a balance in hand of £22 2s. 8d.

### NURSES IN LITIGATION.

There seems to have been a perfect epidemic of nursing litigation lately, and all over the country nurses have been figuring, most undesirably, in the law courts. A particularly indefensible case is reported from the Manchester County Court. The plaintiff, a nurse at Altrincham, sued another nurse trained by her for breaking an agreement made between them that the latter should not carry on "the art, trade, or business," of a nurse within a radius of four miles from the centre of Altrincham, which the plaintiff contended had been done by the defendant attending a case at Peel Causeway last October. Inquiry proved that the defendant, who was nursing in Llandudno, had simply gone to the assistance of a sick friend in Altrincham, staying only so long as her own work permitted, and neither receiving nor expecting fees for her services. The judge, who expressed the opinion that "it was almost as if an injunction had been asked against the Good Samaritan," said the action was most unnecessary and futile and ought never to have been brought, and gave judgment for the defendant, with costs on the higher scale.

### SHORT ITEMS.

The hon. secretary of the Bradford Incorporated Nurses' Institution informs us that it has been decided for the future to grant to each of the private nurses at the end of the year a percentage on her earnings, in addition to the fixed salary.—The nurses of the Glasgow Co-operation for Trained Nurses presented Miss Burgh, their lady superintendent, with a handsome standard brass lamp as a New Year's gift, in "appreciation of her untiring efforts for the success of the co-operation." —On the 13th inst., entertainments for the children took place at the North-Eastern Hospital for Children, Hackney, and were much enjoyed.—The patients at the Forfar Infirmary spent a pleasant Christmas, and an enjoyable concert was got up for their benefit on December 30th.—It is proposed to celebrate the sixtieth year of the Queen's reign at Hampstead by special efforts to help the North London Consumption Hospital, the Hampstead Hospital, and the Hampstead Nursing Association.—An entertainment for the patients and nursing staff of the Chelsea Hospital for Women was arranged for January 12th by the members of the Ladies' Committee, and was a great success.—Mrs. Cameron, the retiring matron of the Princess Alice Memorial Hospital, Eastbourne, has taken over the Brooklyn Institute for Trained Nurses and Surgical Home at 156, Anerley Road, Anerley, S E.—A District Nursing Association, to be affiliated with the Q.V.J.I., is about to be started in Airdrie.

### Hygiene: For Nurses.

By John Glaister, M.D., F.F.P.S.G., D.P.H.Camb., Professor of Forensic Medicine and Public Health, St. Mungo's College, Glasgow, &c.

### XL.—EFFECTS OF OCCUPATION ON HEALTH.

Of the many factors, the sum of which determines the longevity of man, none is so potent as that of his daily occupation. This is true both in relation to the individual and to man in the mass. The ill effects of employment are probably more apparent in these days because of the segregation of workers under entirely artificial conditions, in factories, yards, and workshops. Coupled with the artificial environment, the high-level pressure and competition of the labour market act as a contributing strain on the average workman. Centralisation of population and centralisation of labour have mutually inter-acted upon each other to produce these results. This is especially true of productive industries where the commodity is made out of the raw material. Much less change is apparent in the primitive occupations, as farming, fishing, &c., as these callings can only be followed in the open. It has been alleged that the change which has taken place in the character of each man's labour during the past century has been harmful to the workman. Then the workman was a craftsman who personally undertook all the processes necessary to produce the finished article; nowadays he only takes part in one process or one stage towards its completion. This has reduced the worker from the position of an artist to that of a mere automaton, and the craftsman's pride in beholding the work of his own hands and the relief experienced in the change of incidence of work are thereby lost. However that may be, the variety of occupations to-day is, indeed, very large. It has been computed that there are about 12,000 different occupations, no doubt largely owing to the subdivision of labour.

All occupations may be divided into three classes in respect of the bodily powers drawn upon, viz.: (1) those in which the nervous system is most employed; (2) those in which the muscular system; and (3) those in which both, in varying grades of combination, are used. To the first class belong brain workers generally, as authors, composers, and professional men; to the second the "hewers of wood and drawers of water," and unskilled labourers generally; and to the third skilled workmen generally. It would probably be correct to say that the bulk of occupations belong to this last class. Occupations may be subdivided also in respect of the harmful effects produced by, or incidental to, them. Dr. Ogle gives an excellent working classification on this basis, which we will follow:—

I. Where the workman adopts a cramped or constrained attitude, which prevents the healthy action of lungs and heart.

II. Where he is exposed to the action of poisonous or irritating substances used in the trade.

III. Where he is called upon to work excessively, either mentally or physically.

IV. Where he is compelled to work in confined spaces, and in foul air.

V. Where, from the nature of the occupation, he is liable to contract harmful habits.

VI. Where he is subject to risks to life from accident.

VII. Where he works in atmospheres containing particulate floating matter.

As examples of the first may be cited colliers and miners, clerks, seamstresses, hand-loom weavers, lacemakers (on cushions), glovers, shoemakers (on the last), and workers at benches or machines, in a sitting attitude generally. In all of these, more or less pressure is exerted on the chest, which is often most marked in the case of shoemakers, in whose cases the "last" produces permanent indentation of the breast-bone.

Of the second class, workers in arsenic, mercury, zinc and copper, lead, "chrome," and phosphorus, chemical workers exposed to the fumes of chlorine or sulphur gases, rubber workers to the fumes of carbon bisulphide, and those exposed to the vapours of tar, pitch, or resin, may be taken as examples.

Table I.—Comparative Mortality of Men, 25 to 65 Years of Age, in Different Occupations, 1881-2-3.

| Occupation. | Comparative Mortality. | Occupation. | Comparative Mortality. |
|---|---|---|---|
| Clergymen, Priests, Ministers ... ... | 100 | Builders, Masons, Bricklayers ... ... ... | 174 |
| | — | Carpenters, Joiners ... ... | 148 |
| Lawyers ... ... ... | 152 | Cabinet Makers, Upholsterers ... ... ... | 173 |
| Medical Men ... ... | 202 | | |
| Gardeners ... ... | 108 | Plumbers, Painters, Glaziers ... ... ... | 216 |
| Farmers ... ... ... | 114 | | |
| Agricultural Labourers | 126 | Blacksmiths ... ... | 175 |
| Fishermen ... ... | 143 | Engine, Machine, Boiler Makers ... ... ... | 155 |
| Commercial Clerks ... | 179 | | |
| ,, Travellers . | 171 | Silk Manufacture... ... | 152 |
| Innkeepers, Liquor Dealers ... ... | 274 | Wool, Worsted ,, ... | 186 |
| | | Cotton ,, ... | 196 |
| Inn, Hotel Service ... | 397 | Cutlers, Scissor Makers ... | 229 |
| Brewers ... ... ... | 245 | Gunsmiths... ... ... | 186 |
| Butchers ... ... | 211 | Filemakers... ... ... | 300 |
| Bakers ... ... ... | 172 | Paper Makers ... ... | 129 |
| Corn Millers ... | 172 | Glass Workers ... | 214 |
| Grocers ... ... ... | 139 | Earthenware Makers ... | 314 |
| Drapers ... ... ... | 159 | Coal Miners ... ... | 160 |
| Shopkeepers generally... | 158 | Cornish ,, ... ... | 331 |
| Tailors ... ... ... | 189 | Stone, Slate, Quarriers ... | 202 |
| Shoemakers ... ... | 166 | Cab, Omnibus Service ... | 267 |
| Hatters ... ... ... | 192 | Railway, Road, Labourers | 185 |
| Printers ... ... ... | 193 | Costermongers, Hawkers, Street Sellers ... ... | 338 |
| Bookbinders ... ... | 210 | | |

Table II.—Comparative Mortality from Phthisis and Respiratory Diseases of Men in Various Dust-Inhaling Occupations.

| Men from 25 to 65 years of age. | Phthisis. | Lung Diseases. | Phthisis and Lung Diseases. |
|---|---|---|---|
| Fishermen (as standard) ... .. | 55 | 45 | 100 |
| Carpenters, Joiners ... ... ... | 103 | 67 | 170 |
| Bakers ... ... ... ... ... | 107 | 94 | 201 |
| Wool Workers ... ... ... | 130 | 104 | 234 |
| Cotton ,, ... ... ... | 137 | 137 | 274 |
| Cutlers, Scissor Makers ... ... | 187 | 196 | 383 |
| File Makers ... ... ... ... | 219 | 177 | 396 |
| Masons, Bricklayers ... ... ... | 127 | 102 | 229 |
| Stone and Slate Quarriers ... ... | 156 | 138 | 294 |
| Pottery Makers ... ... ... | 239 | 326 | 565 |
| Cornish Miners ... ... ... | 348 | 231 | 579 |
| Coal Miners ... ... ... ... | 64 | 102 | 166 |

Of the third class, dock labourers, stevedores, athletes generally, porters, engineers, and others are representative. It has been clearly demonstrated that more harm ensues when the work is severe and inconsecutive than where it is steady and continuous. Where the muscular strain is solely confined to certain groups of muscles, as in shorthand writers, pressmen generally, musicians, telegraphists, &c., nerve disturbance, as "writer's cramp," is apt to follow.

Tailors, hatters, bookbinders, printers, machinists, laundresses, shopkeepers, as grocers and drapers, are typical of the fourth class, inasmuch as they work usually in confined, and often foul, air spaces.

Of the fifth class, where vicious habits are contracted from materials used, or products manufactured in the trade—the occupations of innkeeper, brewers and distillers, barmen and barmaids, and liquor dealers generally, carry with them the temptation to contract the alcoholic habit; that of tobacco manufacturer, the abuse of tobacco; and that of druggists, the use of opium, cocaine, ether, or other harmful drugs.

Of the sixth class, little need be said, except that certain occupations are more dangerous from accident than others, such as mining, quarrying, railway workers, or workers generally in large machine shops. To these may be added caisson workers, submarine workers, as divers, and tunnellers under compressed air; and in the making of explosives.

The seventh class embraces a large number of occupations, and the factor, common to them all, which is provocative of harm, is floating matter in the air surrounding the worker, which, when inhaled into the breathing apparatus, produces lung diseases, and, when deposited on the skin, maladies of the skin. The character of the air-particles determines the intensity of the harm produced. The dust particles are either sharp, blunt, or filamentous. In the following occupations are found sharp particles, viz , steel grinders (dry process), stone cutter, sand or glass paper maker, potter, button maker, file maker (by hand), ore miner, quarrier; in the following, blunt particles: wood or ivory turner, sawyer, collier, miller, baker, foundry worker; and filamentous, in those of hemp dressing, flax working, hair and wool sorting, warehouseman, draper, cotton worker, and wool worker. The evil results of substances deposited on the skin are sometimes seen in those engaged in the soot traffic, as in "chimney sweep's cancer," in paraffin workers as skin diseases; and in "chrome" workers, ulcerative disorders.

The mortality arising from any occupation depends upon the following factors, viz.: (1) The intial fitness of the employé; (2) the nature of the occupation; (3) the environment of the employment; and (4) habits of a vicious character which may be contracted by the workman. While it is difficult to attain absolute correctness in mortality rates of occupations, sufficient accuracy is possible to compare the relative incidence of the death-toll. One of the causes of this difficulty is that many occupations, because of their light and easy character, attract the initially weak, disabled, or deformed. This is true of such employments as clerk, tailor, watchmaker, and others. Again, certain other occupations have attractions for men who have saved money in their original occupation, but in which their health has broken down, as inn-keeping or liquor selling. Hence, the mortality rate of a given occupation may not be the result solely of the conditions of that occupation, but partly of that, and of the previously depreciated health or physique.

Of the accompanying tables two which are from Dr. Ogle's paper ("Transactions of Seventh International Congress of Hygiene," Vol. X., pp. 12, *et seq.*) are more eloquent than words. The first table shows the relative mortality of different occupations, and the second the mortality from lung diseases in occupations having different air surroundings; the third table we have compiled to illustrate the various deformities or disabilities which arise in certain occupations.

TABLE III.—OCCUPATIONS WHICH CAUSE PHYSICAL DEFORMITY OR DISABILITY.

| Occupation. | Deformity or Disability. | Proximate Cause. |
|---|---|---|
| Boilermakers, Riveters, or Workers in any concussive noises. | Deafness... ... | Repetitive Concussion of Ear-Drum, &c. |
| Shoemakers ... ... | Indentation or Depression of Breast-bone. | Pressure of Last. |
| Tailors ... ... ... | Peculiar gait in Walking. | Turk-like attitude of lower limbs when at work. |
| Clerks, Students, Scholars, Sedentary Employés generally. | Lateral Curvature of Spine. | Faulty attitude when occupied at work. |
| Telegraphists, Short-hand Writers, Instrumentalists. | Muscular Cramp of Fingers or Hand. | Continuous strained use of Digit-muscles. |
| Clergy (preachers) ... | Affections of Throat and Voice. | Unnatural use of Muscles of Voice-box, and Voice. |
| Miners, Jewellers and Watchmakers, Scientific Instrument Makers, Microscopists, Seamstresses, Engravers | Affections of Eye and Vision. | Strain on Visual Apparatus. |
| Cooks, Gardeners, Car and 'Bus Conductors, Shopkeepers, and persons generally who stand at work. | Varicose Veins and Ulcers of Lower Limbs. | Congestion of veins from effect of gravity and muscular strain on lower limbs. |
| Soldiers, Athletes, Porters, Dock Labourers, &c. | Diseases of Heart, and Large Arteries. | Strain on these organs. |
| Bridge-pier Builders (in caissons), Divers, and Workers under compressed air, Balloonists. | Diseases of Circulation and Nervous System. | Unnatural atmospheric pressure. |

### Death in Our Ranks.

MISS EDITH M. M. SNODGRASS, daughter of the late Major John Snodgrass, 96th Foot, granddaughter of the late Colonel Kenneth Snodgrass, C.B., and of Lieutenant-General Sir Kenneth Douglas, Bart., died at the Citadel Hospital, Cairo, on the 10th inst., after a short illness. Miss Snodgrass entered the nursing profession thirteen years ago, training in the Nightingale School at St. Thomas's Hospital, where she afterwards remained as Sister, her conscientiousness and unusual abilities winning the respect of all. She was declared by Miss Florence Nightingale, who entertained a high appreciation of her capabilities, as "the best night superintendent we ever had at St. Thomas's." Subsequently she entered the Army Nursing Service, and worked successively in the military hospitals of Netley, Gosport, Chatham, and Dublin, having charge of the enteric fever wards for five years in the last-named place. In October last her turn came for foreign service, and she was ordered to Egypt for duty at the Citadel, Cairo. Miss Snodgrass possessed talents of no ordinary degree, and these she devoted to her work. She was a skilful and excellent nurse, and ungrudgingly gave her strength and loving care to those who needed them. The nursing service has in Sister E. Snodgrass lost one whom it could ill spare, and whose memory will ever be cherished with love and reverence. Of her it may be truly said that she gave her life "to works of mercy among the sick and suffering," and to anything which might "tend to brighten the lives and ameliorate the condition" of the poor.

### Midwifery Papers.

### XII.—PUERPERAL PERIOD: GENERAL MANAGEMENT.

The time which elapses between the birth of the child and the complete convalescence of the mother is called in midwifery the puerperal period. Immediately after delivery the uterus contracts, but some weeks elapse before it regains its normal size and recedes into its normal position in the "true" pelvis. Its decrease in size begins to be apparent twenty-four hours after delivery. The fundus can be felt about two inches above the pubes on the ninth day, and generally, at the end of the fortnight, it has quite disappeared behind the pubes and can no longer be felt abdominally.

After-pains.—The intermittent and generally painful contractions of the uterus which continue for some days after the confinement are called "after-pains." They are useful in expelling clots which may be retained in the uterus, and are said by some authorities to be excited by such clots. They assist the reduction in size of the uterus by contracting the enlarged muscles and blood-vessels, and so diminishing its blood-supply. "After-pains" are not so severe in "first" cases, where the uterus contracts more powerfully immediately after delivery than it does in the case of women who have borne many children, and where the uterine-walls are weakened and relaxed by frequent distention.

Temperature and Pulse.—During the intense muscular exertion of the latter stage of labour the patient perspires freely, and directly labour is over an extra blanket should be thrown over her, for the surface temperature falls rapidly, and she may easily take a chill. If the latter stage has been severe and prolonged, the temperature rises, and it may remain a degree or two above normal till the patient has been refreshed by sleep. The pulse is slowed after labour, and sometimes falls to 50. This sub-normal rate may continue for a day or two. If after labour the pulse rises to 100, extra watchfulness is necessary, as it may be a danger-signal of hæmorrhage. Observations of both pulse and temperature should be taken for the first 14 days, night and morning, for during that period they are apt to vary. They are easily affected by slight nervous or physical changes, but sudden rises should be watched carefully, as they may be due to something more serious—as septic absorption or other mischief. There is sometimes a rise of temperature on the third day, accompanying the onset of the milk, especially if the secretion is great and the breasts become swollen and knotty. The temperature subsides when the fulness is relieved. Constipation also sends the temperature up.

Excretions.—After the confinement the bowels are generally constipated, and an enema of soap and water should be given on the third day. If this is properly given, it disturbs the patient less than a dose of castor oil. After this a daily action of the bowels should be secured. During the first few days the patient generally passes an abnormal quantity of water. The patient should be encouraged to pass water an hour or two after labour. Sometimes there is a difficulty owing to some paralysis, from pressure or from bruising of the parts during labour. And many nervous patients find the horizontal position over a bed-pan a great difficulty in passing water. If the difficulty is only due to nervousness a little tact and patience on the nurse's part may help to overcome it. The patient should never be allowed to sit up on a bed-pan till after the first twelve hours, and only then if there has been no sign of hæmorrhage. Her back should be well supported by pillows. If difficulty in passing water persists, the nurse may relieve it by hot fomentations over the bladder or by sponging the vulva with warm water. If the parts are bruised and sensitive, the fomentations to the vulva should not be too hot. As a last resource, the catheter should be passed and the water drawn off, for should the bladder become distended it is not only exeeedingly painful to the patient, but dangerous, as its prevents uterine contraction by its pressure. Before passing the catheter it should be washed in a solution of carbolic or mercury and annointed with vaseline or carbolic oil. In midwifery cases the catheter should be one which has not been used before. The vulva should be carefully sponged with a warm solution of carbolic ($\frac{1}{50}$) or mercury ($\frac{1}{1000}$) to cleanse it from all discharge before introducing the catheter, so that there shall be no chance of conveying septic matter into the bladder. Every care also should be used in passing the catheter not to hurt the parts which may be already bruised and painful.

The Lochia.—The uterine discharge, which goes on for the first two or three weeks of the puerperal period, is called the lochia. During the first three or four days it is more or less sanguinous, and blood-clots varying in size are passed. Shreds of decidua are also to be seen in it. The discharge loses its bloodlike appearanee after the fourth day and becomes paler, and towards the ninth day it has a slightly greenish colour. In most cases the lochial discharge varies in amount; some women have an abundant flow, while in others it may be quite scanty. The lochia have an unpleasant odour, and quickly decompose after leaving the vagina. Bed and body linen soiled by this discharge should be changed and at once removed from the room. If the patient lies too long on her back or side the lochia are often retained in the uterus or vagina, so she should be encouraged to change her position in bed from time to time, as this assists the escape of the discharge. An injection of Condy's fluid night and morning prevents the accumulation of discharge within the vagina. It is a good plan to bathe the external genitals with a warm antiseptic three or four times a day, if the discharge is profuse and the napkins require frequent changing. Too much care cannot be exercised to keep the patient and all her surroundings perfectly clean. If the lochia are very profuse, and the parts are not frequently cleansed, they are apt to get sore, and cause much discomfort to the patient. If there is any soreness or rawness of the external genitals, eucalyptic vaseline applied gives relief. This should be applied after the parts have been cleansed from all discharge.

The Breasts.—For the first two days after the birth the breasts secrete a thin, clear, slimy fluid called colostrum. The child should be put to the breast two or three times a day during the secretion of the colostrum, as it has an aperient effect which is of service to the new-born infant. On the third day the secretion changes its character, and the breasts become full of milk and are sometimes swollen and sensitive and knotty, especially if the nipple is cracked or painful or retracted, and the child is not able to suck freely. If flat, the nipple may be gradually drawn out by using the breast-pump, and tension may be relieved in this way by drawing off a little of the milk. If for some reason or other the mother cannot suckle her child and the milk is to be suppressed, the breasts should be supported and compressed by bandages, the nipples being left free. If the breasts are painful paint them with a mixture of belladonna and glycerine, and apply hot fomentations and then bandage. When the milk is to be got rid of the breasts should never be drawn or rubbed, as this stimulates the gland and excites secretion. Care should be taken from the first to prevent a great accumulation of milk, for this gives the patient a great deal of pain, and often leads on to inflammation and suppuration. After suckling the nipples should be washed with a little warm water and carefully dried to prevent soreness or crack. The child should be put to each breast in turn to relieve any tension and encourage equal secretion of milk.

DIET.—For the first two or three days after labour the patient's diet should be of a light and easily digestible character. Beef-tea or soup, milk and milk gruel, tea or coffee, a lightly boiled or poached egg, thin bread and butter or toast, may be given at intervals. On the third day afterwards the bowels have been moved, fish or chop may be given. In all cases of continued raised temperature a liquid diet should be adhered to till the temperature subsides. In most cases the appetite varies, and the patient's taste should be consulted as far as possible, especially if the appetite is at all capricious. If the mother is suckling the child an ampler diet with a fair amount of milk is required. If the milk is to be suppressed the woman will not need so much food, and she should be told to drink as little as possible. It cannot be too much impressed on nursing mothers that milk forms a necessary part of their diet, and is far more nourishing than ale or porter, which so many women consider so exceedingly necessary at this time. The diet of the mother has a great effect on the quality of the milk, and during the period of lactation the simpler the diet is the better for the nourishment of both mother and child. Rich or highly-seasoned foods and alcohol in any form are apt to change the character of the milk and often upset the digestion of the child. The mother should remember this, and deny herself all unsuitable diet.

GENERAL MANAGEMENT.—The patient should be kept in bed for the first ten days, if possible. This is often difficult to manage among the poorer classes, and many poor women get up and return to their hard duties long before they are fit, and so lay the seeds of much after-suffering. On the tenth day the patient should be allowed to leave her bed for the sofa, and each day following she should be allowed to take a step forwards towards a more active life. On the fourteenth day, if good progress continues to be made, she may be allowed to leave her room, and, a day or two after, if the weather is fine, a drive or short walk may be taken. If the discharge becomes sanguinous, or has not lost its red colour, the patient should remain longer in bed, and should not be permitted to undertake much exertion. If there has been any laceration or severe hæmorrhage at the birth the recumbent position should be retained for a week or two after the usual time. The uterus does not regain its normal condition until six weeks after the birth of the child, so during that period no great muscular exertion should be undertaken.

A nursing mother should carefully conform to all rational laws of health. She should take daily walking exercise in the open air, if possible, and she should lead an active life free from unhealthy excitement. Tight corsets and unsuitable clothing of all kinds should be avoided; she should wear warm, light, loose clothing. During lactation the health of the infant is dependent on the health of the mother, and mothers should not readily forget this.

### Mr. Hall Caine at Sea.

### A NURSING NIGHTMARE.

THE poor trained nurse! Criticised in all seriousness in the columns of the *Nineteenth Century*, the *National Review*, the *Practitioner*, the *Queen*, she is now being held up to ridicule—in apparent unconsciousness, too—in the new serial story by Mr. Hall Caine in the pages of the *Windsor Magazine*. And very sorely and righteously aggrieved she has every reason to feel at this last injury, for it is a great injury when an author with the reputation of Mr. Hall Caine depicts in a most objectionable, and entirely untrue, light people and places with which he has evidently no acquaintance at all. In the first chapters of "The Christian," wherein Glory Quayle, Mr. Hall Caine's heroine, becomes a probationer at "the Hospital of Martha's Vineyard, Hyde Park, London," there is sufficient ignorance of detail shown to convict an author of very great carelessness, to say the least, but the climax is reached in the current number, in which is given a highly coloured and purely imaginary description of a ball at "Bartimæus's Hospital," given by the hospital authorities "to all the nurses of London who do not happen to be on night duty." And the ball is held in "the operating theatre of the hospital—a great circular hall, with a gallery running round its walls, which were now festooned with flags and roofed with a glass dome, from which coloured lamps were hanging!" But this is not all; in this wonderful "operating theatre" (who can have been priming the writer with fairy tales?) "some four hundred girls, and as many men, were gathered there; the pit was their dancing-ring, and the gallery was their withdrawing-room!" This hypothetical scene is, moreover, actually illustrated to represent something like the interior of the Albert Hall, so that on the face of it, neither author nor artist can ever have been inside a hospital, or they would have had some approximate knowledge of the size of such a place. The President of the College of Surgeons opens the ball, by the way, but presently he, "the great doctors and the matrons," leave the scene of revelry, "only the nurses and the students remained, and the fun was becoming furious; somebody lowered the lights and they danced in a shadowland; somebody began to sing, and they all sang in chorus. . . ." It was three in the morning when the ball broke up, Glory and another probationer being escorted back to "Martha's Vineyard" by two men of their acquaintance, to find the hospital chaplain, a friend of Glory's, waiting for her in the hall, when a "peal of noisy laughter" draws the attention of a "ward sister going by at the moment." Instances of other equally glaring absurdities might be enumerated, and the whole thing is such a travesty of fact that it would be exceedingly funny if it were not for the thought of the harm such fiction does when read by those who know no more of the matter than does the author. Mr. Hall Caine has roused a great deal of well-deserved anger and indignation amongst a body of women whose good work should have protected them from such treatment as that meted out to them by him. At least, if he values his own reputation, an apology is due to nurses no less than to hospitals for the caricature now being perpetrated at their expense in the *Windsor Magazine*. The illustrations, wherein fast and objectionable women are pictured in nurses' uniform, are a crowning offence to every self-respecting member of the nursing profession.

### Where to Go.

LONDON HOSPITAL, WHITECHAPEL ROAD.—On Monday, February 1st, Mr. Bancroft will give a reading of Charles Dicken's "Christmas Carol" in the library of the London Hospital Medical College, the entire proceeds to go to the hospital funds. Tickets 5s. each, to be had from the secretary at the hospital.

TEMPERANCE HOSPITAL.—A musical cantata, performed by children, will be given, by request, in aid of the above hospital on Monday, February 1st, at Emmanuel Hall, Harrow Road; to commence at eight o'clock. Tickets, 1s. (reserved) and 6d., may be obtained beforehand from Miss Herbert, 27, Charleville Road, West Kensington, W.

### Everybody's Opinion.

[Correspondence on all subjects is invited, but we cannot in any way be responsible for the opinions expressed by our correspondents. No communication can be entertained if the name and address of the correspondent is not given, or unless one side of the paper only is written on.]

### THE MEDICO-PSYCHOLOGICAL ASSOCIATION AND ITS NURSES.

Miss B. Jones writes from Berrywood Asylum: Referring to Miss Wingfield's and to other letters and articles on the above subject, I think that it is misleading and unfair to the trained mental nurse to say "it would degrade the register of the R.B.N.A." to admit properly trained mental nurses to it. I have been trained in this asylum and have obtained my certificate as a trained mental nurse, this being, so far as I know, the only institution of its kind which instructs, trains, and examines its own nurses precisely on the hospital system. Here it is necessary for each nurse to go through a period of probation in the sick ward, and to attend in this asylum three years' courses of lectures and instructions. My fellow nurses and I think we are on the same level as those nurses who have gone through a three years' training in a general hospital; we might even claim to be on a higher level, because we understand mental nursing as well as sick nursing. We have not yet asked to be placed on the register of the R.B.N.A., but we cannot see how we could "degrade" it.

Dr. T. Outterson Wood writes: I have to thank you for having published my letter, and I must ask you to kindly permit me to answer your editorial remarks. I have told you that no candidate can pass the examination of the Medico-Psychological Association without giving evidence of an intimate knowledge of practical nursing in its various branches. To this you say I miss the point at issue! Is this "cramming out of a text-book?" You go on to say, "No woman can be called a trained nurse who has not had practical experience in the work of the sick room. Occasional demonstrations cannot take the place of this. Some of the medico-psychological nurses have had this training, others have not." Pardon me, you are wrong again. In every asylum or hospital for the insane there are the hospital wards where the sick patients are nursed and cared for as well and as scientifically as they are in a general hospital. The nurses in charge of these wards, whenever practicable, have had hospital training. Through these wards the candidates for the medico-psychological certificate for "proficiency in nursing" pass. But this is not all. There are, in addition, a large number of paralysed, demented, and helpless cases which must be carefully nursed, fed, and treated, but for whom there is not accommodation in the hospital wards—besides the acutely maniacal and melancholic who are liable to illnesses and injuries of all kinds, and who must be nursed, so that the sick-room experience of these mental nurses is necessarily and compulsorily a large one. They cannot pass their examinations without it. Now, sir, whether you or I approve of the title "mental nurse" or not, it exists; it is in general use by the medical profession, and by the public, and I claim that those who hold the certificate of the Medico-Psychological Association have a right to tnat title in its highest and best sense. In justice to themselves, and for the protection of the public, they have a right to demand that their title shall be registered, and I hold that the proper place for it is in a mental department of the published register of the R.B.N.A.

### LADY PRIESTLEY AND THE NURSES.

A Private Nurse writes: With regard to Lady Priestley's unmerited attack on nurses in her article to the *Nineteenth Century* I should like to say that though I do not know what ground of offence she has against us, it is a pity she cannot change places with us for a while and see for herself some of our drawbacks and hardships. There is in private nursing a good deal more than meets the eye. It does not all consist of "dancing about bachelors' apartments" in costumes not altogether unbecoming (becoming only by reason of their cleanliness and simplicity, which many ladies would do well to imitate), and "goings on" with gentlemen in public watering places. Let her experience the misery of entering a stranger's house as professional nurse, the sinking of heart with which one starts for a case, one's courage getting lower and lower with each turn of the cab-wheels, each stoppage of the train, never knowing how you will be received, and what you are going to encounter—the long night watches, the suffocating smells of the sick-room, the whims and vagaries of patients, and what not. Of the "jealous wives" I cannot speak with too great a contempt—they cannot evidently do without us, or they would not have us, and it behoves them to treat us properly; we want nothing of their husbands. It is only natural that invalids should like best to be waited on by those who understand how to handle them, as most untrained women, in spite of frequent assertions to the confrary, are of little use in the sick-room. With regard to the fee, the labourer is worthy of his hire, and we have a right to our proper fee just as much as doctors and other professional people have to theirs. I think Lady Priestley overlooks the fact that nurses have not many working years, and that no nurse who is on her own account can afford to work for a guinea a-week. I should like it to be understood that private nurses work simply and solely for money, in spite of things that have been said to the contrary. There is no reason why a nurse should not work for money just as a doctor or a clergyman does. Our working for money does not prevent our working wisely and well, indeed I have no doubt we should not work nearly so well were we not paid for it. The suggestion that we should be bound by vows and belong to a sisterhood is absurd on the face of it. In those countries in which the nursing is done by religious sisterhoods, notoriously in France, the nursing is very inferior, and training in our sense of the word is not even attempted. The very dress of the sisters is cumbersome and inconvenient, and irritating to patients. And why, pray, are we, many of us young women, because our duty calls us to watch over the last moments of the dying, to be debarred from participation in the pleasures of life? A doctor, I presume, or even an undertaker, would be allowed to indulge in pleasures galore! It is so wrong to judge of a hard-working community by one or two members of it, or to condemn a whole flock because of a couple of black sheep which belong to it. I am sorry that the views expressed in The Hospital should be rather in favour of these tirades. I am sure, in these days, when nurses are so largely employed, they ought to receive all support and sympathy from the public, leading as they do a peculiarly trying life.

*** If the author of this letter will read our remarks again we hope that she will see that we are not in favour of "these tirades." What we point out is that in the present state of affairs a certain number of women are drawn to nursing who turn out to be unfitted by nature ever to become first class nurses of the stamp that is understood to be expressed by the term "trained nurse." It is, however, out of the question to turn such people out into the cold when they have given some years of their life to the acquisition of such knowledge as they possess, and what we submit for the consideration of nurses is whether it would not be best to "grasp the nettle," to accept the facts as they exist, and admit a second class even in nursing. We think that there is field enough for a very large extension of private nursing if a nurse could be provided at a lower fee, even though not quite so competent as are the better sort of the fully-trained nurses of to-day. We do not want, however, to lower by one iota either the training or the status of the "trained nurse," knowing well how necessary the highest nursing skill is in serious cases. But all cases are not serious, and we do not care to "peel potatoes with a silver knife." To get over the difficulty, and meet a growing demand, we threw out the suggestion of a second class. Will any one suggest a better way?—Ed. *T. H.*

### Wants and Workers.

A lady is anxious to know of any well-recommended home where a poor but very respectable man, suffering from chronic chest affection (not consumption), could be received in payment of 8s. or 9s. a week. West of England preferred.

### For Reading to the Sick.

"FRET NOT THYSELF."

**Verses.**

I SOUGHT for Peace, but could not find;
I sought it in the city;
But they were of another mind—
The more's the pity.

I sought for Peace of country swain,
But yet I could not find;
So I, returning home again,
Left Peace behind.

Sweet Peace, where dost thou dwell? said I;
Methought a voice was given,
"Peace dwells not here, long since did fly
To God in Heaven."

Thought I, this echo is but vain,
'Tis folly 'tis of kin;
Anon I heard it tell me plain,
'Twas killed by sin.

Then I believed the former voice,
And rested well content;
Laid down and slept, rose, did rejoice,
And then to Heaven went.

There I enquired for Peace, and found it true;
An heavenly plant it was, and sweetly grew.
—*Anon.*

Lord, as to Thy dear cross we flee
And plead to be forgiven,
So let Thy life our pattern be,
And form our souls for heaven.

Help us, through good report and ill,
Our daily cross to bear;
Like Thee to do our Father's will,
Our brethren's grief to share.

Kept peaceful in the midst of strife,
Forgiving and forgiven,
O may we lead the pilgrim's life,
And follow Thee to heaven!
—*J. Hampden-Gurney.*

There is a land of peace,
Good angels know it well,
Glad songs, that never cease,
Within its portals swell;
Around its glorious throne
Ten thousand saints adore
Christ, with the Father one
And Spirit evermore. —*H. W. Baker.*

**Reading.**

O, that thou hadst hearkened to My commandments! Then had thy peace been as a river, and thy righteousness as the waves of the sea.—*Isaiah* xlviii. 18.

Oh, no; you will ever be a stranger to true peace; you will know nothing of true happiness until you find it at the foot of the Cross—in a sense of pardon, reconciliation, and acceptance with God.—*Winslow.*

Is it any use to read this Psalm ("Fret not thyself") in days when you are racked with a continual fever of frettint and nerves, and anxieties, half of which you know (b u cannot feel) to be groundless?

Our Lord once took a woman's hand and "the fever left her." If He took your hand, this worst fever would leave you —this fever of the nerves which is wearing you to threads. Is it your fault, or His, that He does not do so?

That woman "arose and ministered to them." How you envy her! But it is not your bodily illness which hinders you from ministering, half so much as that spiritual fever of a rebellious will. Others, who had more weakness and worse pain to bear, have been helpful members of those households, because they accepted God's will peacefully—and nothing would so help your household as to see this peace growing in you. . . . It is not impossible to feel the Master's Hand; and His touch calmed them that were possessed with blacker spirits, deeper depression, than you have ever known.—*L. H. M. Soulsby.*

### Appointments.

MATRONS.

CITY OF OXFORD INFECTIOUS DISEASES HOSPITAL.—Miss Janet Hardisty has been appointed Matron at this hospital. She was trained at the Liverpool Royal Infirmary, and has since worked at the Bristol Royal Infirmary and Hull Sanatorium.

HARLINGTON, HARMONDSWORTH, AND CRANFORD COTTAGE HOSPITAL.—Miss Ankritt has been appointed Nurse-Matron at this hospital. She was trained at the Cheltenham General Hospital, afterwards taking six months' work in a fever hospital. Miss Ankritt has also had some experience in district and private nursing.

### Novelties for Nurses.

BUDGET LETTER CARDS.

MESSRS. LANGLEY AND SONS, of George Street, N.W., have sent us specimens of their new court-shaped budget letter-cards. These cards are a distinct improvement on any we have hitherto seen. The edges are rounded and less liable to be torn, whilst the general appearance is distinctly superior to that of the cards mostly used.

### Notes and Queries.

**The contents of the Editor's Letter-box have now reached such unwieldy proportions that it has become necessary to establish a hard and fast rule regarding Answers to Correspondents. In future, all questions requiring replies will continue to be answered in this column without any fee. If an answer is required by letter, a fee of half-a-crown must be enclosed with the note containing the enquiry. We are always pleased to help our numerous correspondents to the fullest extent, and we can trust them to sympathise in the overwhelming amount of writing which makes the new rules a necessity. Every communication must be accompanied by the writer's name and address, otherwise it will receive no attention.**

*Workhouse Nursing.*

(96) Please tell me if trained nurses can join the Workhouse Infirmary Nursing Association without leaving their present work?—*Sister May.*

Write to Miss Gill, Workhouse Infirmary Nursing Association, 6, Adam Street, Strand, London, for particulars.

*Midwifery Papers.*

(97) Can you tell me if the midwifery papers published in THE HOSPITAL will be brought out in book form, and if so, where I can get them?—*A Reader.*

It is not at all probable that those articles will be republished in book form.

*Training.*

(98) Would a Jewess be admitted as probationer into any of the London hospitals, keeping her own religion?

No religious distinctions are made in most London or other hospitals in these days. The London Hospital, Whitechapel Road, is perhaps one of the most unsectarian.

*Nursing Home Wanted.*

(99) Can you tell me of a nursing home at the seaside, Margate preferred, where a gentleman could be taken who is wearing a Thomas's splint and suffering with his hip?—*A. C.*

Write to Nurse Bissell, Lynton Villa, Cliftonville, Margate.

*Health Lectures.*

(100) I have been asked to give some health lectures to members of the Y.W.C.A. in the parish where I am district nurse. Can you advise me as to books to study in preparation for a simple course of such lectures?—*Sister Henrietta.*

Dr. Percy Lewis's "Theory and Practice of Nursing," and Mrs. Dacre Craven's "Guide to District Nurses and Home Nursing" have been found most helpful by nurses who have given such lectures. You can get both books from the Scientific Press, 28 & 29, Southampton Street, Strand, W.C.

*Junius S. Morgan Benevolent Fund.*

(101) Can you tell me the address of the "Nurses' Benevolent Society"?

Do you mean the Junius S. Morgan Benevolent Fund for Nurses? Write to the hon. secretary, Miss Rosalind Pritchard, 82, Talbot Road, W.

*Polished Floors.*

(102) What is the best method of polishing a ward floor already stained and varnished?—*R. R.*

Read the methods of treating floors described in THE HOSPITAL of December 5th, 1896, page 171. Linseed oil, with a little ol. eucalyptus added, is there recommended as being better and cheaper than beeswax and turpentine for polishing stained floors.

